# The comparison of two different mandibular positions for oral appliance therapy in patients with obstructive sleep apnea

**DOI:** 10.1002/cre2.650

**Published:** 2022-08-23

**Authors:** Eri Makihara, Takafumi Watanabe, Hiromichi Ogusu, Shin‐Ichi Masumi

**Affiliations:** ^1^ Division of Occlusion & Maxillofacial Reconstruction, Department of Oral Function Kyushu Dental University Kitakyushu Japan

**Keywords:** mandibular position, obstructive sleep apnea, oral appliance, treatment effectiveness

## Abstract

**Background:**

An oral appliance (OA) can alleviate upper airway obstruction by pulling the mandible forward during sleep. While a large mandibular advancement with an OA decreases the number of apnea and hypopnea events, long‐term use may cause side effects, such as toothache, stiffness, and pain in the temporomandibular joint.

**Objetives:**

This study aimed to evaluate the effectiveness of different mandibular positions for obstructive sleep apnea (OSA) and determine the optimal therapeutic mandibular position.

**Methods:**

Thirty‐two patients (17 males and 15 females) with mild to moderate OSA participated in this prospective study. All patients were randomly allocated to receive a 50% mandibular advancement or a 75% mandibular advancement with an OA. The pre‐ and posttreatment apnea‐hypopnea index (AHI), apnea index (AI), and Epworth Sleepiness Scale (ESS) were compared. Treatment effectiveness and treatment success were compared between groups.

**Results:**

AHI improved significantly in both groups, and AI improved significantly in the group with 50% mandibular advancement. No significant improvements in the ESS were observed in either group. There was no significant difference in treatment effectiveness between groups. In the proportion of females and males whose treatment was effective in the two groups, females were significantly greater than males.

**Conclusions:**

For patients with mild to moderate OSA, 50% mandibular advancement is recommended as the initial therapeutic mandibular position. It was suggested that gender differences also affect treatment effectiveness.

## 1 INTRODUCTION

Sleep apnea has been increasingly recognized as an important health concern in recent years. This has been particularly the case for obstructive sleep apnea (OSA), which is an intermittent apnea that occurs during sleep and is a possible trigger for diseases, such as hypertension (Cui et al., 2008), diabetes (Al‐Delaimy et al., 2002), and cardiovascular diseases (Gami et al., 2004; Redline et al., 2010; Schäfer et al., 1999). In OSA, upper airway muscle such as genioglossus muscle activity decreases during sleep same as other skeletal muscles (Wheatley et al., 1993), and the base of the tongue and soft palate move posteriorly, thereby blocking the airway and causing symptoms such as decreased oxygen saturation in the blood and the inability to obtain satisfactory sleep (Phillipson, 1993; Strohl et al., 1986). OSA treatment includes continuous positive airway pressure (Sullivan et al., 1981), surgery (Fujita et al., 1981; He et al., 1988; Li et al., 2002; Powell et al., 1999), as well as conservative management, such as weight loss (Strobel & Rosen, 1996) and the provision of an oral appliance (OA) by dentists (Ramar et al., 2015). An OA can alleviate upper airway obstruction by pulling the mandible forward during sleep.

We have previously investigated the long‐term use of OAs in 48 OSA patients. Patients who continued to use their OAs exhibited improved polysomnography (PSG) results and fewer subjective symptoms. However, hypersalivation, dry mouth, and pain and discomfort in the temporomandibular joint were reported as side effects (Makihara et al., 2016). The most common reasons for OA discontinuation were the associated side effects and the lack of treatment effectiveness (Makihara et al., 2016; McGown et al., 2001).

At present, therapeutic mandibular positions are used in 50%–89% of OA treatments for OSA (Bartolucci et al., 2016). For OA treatment to be effective, the mandibular position must be close to the most protrusive position. However, maintaining such a position over long periods may cause side effects, such as toothache, stiffness, and pain in the temporomandibular joint (de Almeida et al., 2002). Previous studies have reported associations between low compliance with OAs and both side effects and a lack of treatment effectiveness (de Almeida et al., 2002). Therefore, the incidence of OA‐associated side effects needs to be reduced in order to facilitate the continued use of OA therapy in OSA patients. This study aimed to compare the effectiveness of OSA treatments using two different mandibular positions and determine the optimal therapeutic mandibular position.

## 2 MATERIALS AND METHODS

### 2.1 Subject selection and recruitment

This was a prospective study, and the protocol was approved by the Kyushu Dental University Institutional Review Board (Approval on October 31, 2012, Approval No. 12‐18). The subject selection criteria were adult patients with mild to moderate OSA (apnea‐hypopnea index [AHI] ≥ 5 and <30), who underwent overnight PSG at nearby medical institutions, and were subsequently prescribed OA treatment. OAs were provided at the authors' institution, from August 2013 to October 2015. The details of this study (purpose, period, number of visits, items to be examined, and measurement method) were explained in detail to all patients. Exclusion criteria included the following: OSA patients who were being treated with methods other than OAs; central sleep apnea patients with a central apnea index (AI) > 5; and patients with psychiatric disorders, other sleep disorders, orofacial abnormalities, or multiple missing teeth. Thirty‐two patients (17 males and 15 females; mean age: 62.2 ± 1.90 years old) were recruited and provided written informed consent.

Daytime sleepniness data using Epworth Sleepiness Scale (ESS) (Johns, 1991) were collected from the patients; before and after 3‐4 months from the start of OA treatment.

OA treatment was deemed to be effective if AHI improved to 50% or higher, and posttreatment AHI was less than 10 (Almeida et al., 2009; Krishnan et al., 2008; Kuna et al., 2006). The mean pretreatment AHI in patients with OSA (who visited our clinic between January 2007 and September 2012) was 22.3 ± 13.49. Based on this data, the AHI was anticipated to improve to approximately 56%, with a posttreatment AHI < 10. The null hypothesis was that posttreatment AHI was the same as pretreatment AHI, and the alternative hypothesis was that posttreatment AHI was significantly different from pretreatment AHI. The sample size calculation was based on the following parameters: (1) effect size of 56% × 22.3 = 12.49; (2) standard deviation of pretreatment AHI = 13.49; (3) standard effect size = 0.925; and (4) α (two‐sided) = 0.05，and power (1‐β) = 0.20. Therefore, 14–16 OSA patients were required in each group (Browner et al., 2007).

### 2.2 Fabrication of OAs

OSA patients were randomly allocated to receive a 50% mandibular advancement or a 75% mandibular advancement using the envelope method. Using the George gauge (George, 1992), the amount of mandibular adjustment was measured at a distance of 2 mm between the upper and lower incisors. After measuring each therapeutic mandibular position at 50% and 75% mandibular advancement, the OA was constructed using the TheraSnore™ (DISTAR, Albuquerque) and provided to the patients. The OA was a boil‐and‐bite appliance, which could be separated through the snap mechanism attached to the top and bottom. The mandibular position could be adjusted by 1.5 mm at five levels. However, in this study, the subjects were instructed not to change the initial therapeutic mandibular position. Following the provision of the OA, the patients were reviewed at the hospital once every 4 weeks. They were assessed for pain in the oral mucosa and teeth. If pain was present, the inner surface of the device at the corresponding site was grounded and adjusted. Pain in the temporomandibular joint and masticatory muscles was also assessed in accordance with previously established diagnostic criteria for temporomandibular disorders (Schiffman et al., 2014). Furthermore, the posterior teeth contact was assessed by avulsion test using articulating paper every visiting time. Moreover, patients were instructed to remove the OA after waking up the next morning, bring the posterior teeth back into the intercuspal position, and massage the masticatory muscles.

### 2.3 Assessment of effective treatment

After 3–4 months from the start of OA treatment, we assessed the usage frequency of the appliance, and whether the patients could use the appliance for at least 5 days a week, and once every 4 h. The PSG test was performed at the original referring medical institutions. The pre‐ and posttreatment AHI, and the post‐ and pretreatment AI of 28 subjects were compared. Pre‐ and posttreatment subjective evaluations were carried out among 15 subjects using the ESS. The treatment was deemed “effective” (Almeida et al., 2009; Krishnan et al., 2008; Kuna et al., 2006) if the posttreatment AHI improved to < 10 and the AHI improved to 50% or higher. The treatment was deemed “slightly effective” if the sufficient therapeutic effect was not achieved, and “aggravated” if the posttreatment AHI was higher than the pretreatment value. Furthermore, treatment success was defined by the following criteria: posttreatment AHI improved to < 10, and posttreatment AI improved to < 5 (Van Haesendonck et al., 2016).

### 2.4 Statistical analysis

All statistical analyses were performed using SPSS for Macintosh version 27.0 software (SPSS). The Shapiro‐Wilk test was used to assess the normality of the distribution of data. The Pearson's chi‐squared test was used to compare treatment success between groups, as well as the proportion of females and males, with treatment success in both groups. Significant differences in mean values between the two groups were assessed using the parametric Student's unpaired *t*‐test and nonparametric Mann–Whitney *U*‐test. Significant differences in mean values between the pre‐ and posttreatment variables were assessed using the nonparametric Wilcoxon signed‐rank test. In all cases, a significant difference was set at *p* < 0.05, which was two‐tailed.

## 3 RESULTS

**Figure 1:**
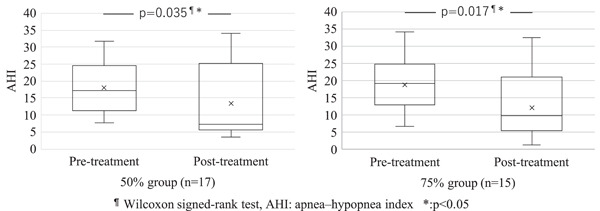
The change in AHI after treatment in the two mandibular advancement groups. Significant differences were observed between pre‐ and posttreatment AHI in both groups.

**Figure 2:**
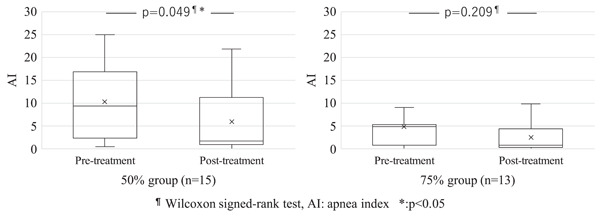
The change in AI after treatment in the two mandibular advancement groups. A significant difference was observed between pre‐ and posttreatment AI in the 50% mandibular advancement group.

**Figure 3:**
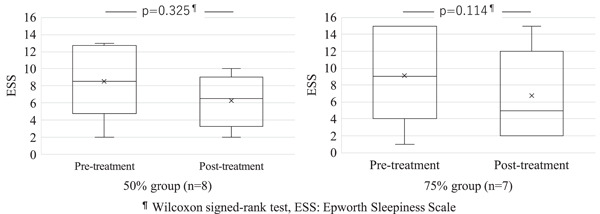
The change in ESS after treatment in the two groups. No significant differences were observed between pre‐ and posttreatment ESS in either group.

No significant differences were observed between the two groups for age, sex, body mass index, and both pretreatment and posttreatment ESS, AHI, and AI (Table 1). AHI improved significantly in both groups (Figure 1). The AI data of each two persons in two groups from some medical institutions were lacking. Although there might be some reasons of data management on the medical institutions, the exact cause was unclear. AI improved significantly in the group that underwent 50% mandibular advancement (Figure 2). No significant improvements in ESS were observed in either group (Figure 3).

**Table 1:** Comparison of patient characteristics between groups

Characteristics	50% group (*n* = 17)	75% group (*n* = 15)	*p*
Gender (male/female), *n*	8/9	9/6	0.502^a^
Age, years, median (IQR 25–75 percentile)	70.0 (60.0–72.0)	61.0 (54.0–65.0)	0.316^b^
BMI (kg/m^2^), mean ± SD	23.79 ± 3.24	23.72 ± 2.80	0.950^c^
Pretreatment AHI, median (IQR 25–75 percentile)	17.10 (12.00–22.22)	19.20 (13.55–24.40)	0.823^b^
Posttreatment AHI, median (IQR 25–75 percentile)	7.20 (5.60–24.40)	9.90 (5.70–18.25)	0.955^b^
Pretreatment AI, median (IQR 25–75 percentile), *n*	9.30 (3.40–15.90), 15	4.80 (0.90–5.20), 13	0.080^b^
Posttreatment AI, median (IQR 25–75 percentile), *n*	1.70 (1.00–7.70), 15	0.80 (0.50–3.60), 13	0.213^b^
Pretreatment ESS, median (IQR 25–75 percentile), *n*	8.50 (6.25–12.25), 8	9.00 (6.50–13.00), 7	0.780^b^
Posttreatment ESS, median (IQR 25–75 percentile), *n*	6.50 (3.75–9.00), 8	5.00 (3.50–9.00), 7	1.000^b^

*Note*: No significant differences in patient characteristics were observed between the two mandibular advancement groups. 50% group: 50% mandibular advancement; 75% group: 75% mandibular advancement. Abbreviations: AHI, apnea‐hypopnea index; AI, apnea index; BMI, body mass index; ESS, Epworth Sleepiness Scale; IQR, interquartile range; SD, standard deviation.

^a^ Pearson's Chi‐squared test.

^b^ Mann–Whitney *U*‐test.

^c^ Student's unpaired *t*‐test.

Among patients who underwent 50% mandibular advancement, treatment was effective, slightly effective, and aggravated in eight subjects (47.1%), five subjects (29.4%), and four subjects (23.5%), respectively. Among patients who underwent 75% mandibular advancement, an equal number of patients (*n* = 5, 33.3%) were documented in each of the three treatment effectiveness categories. No significant difference in effective treatment was observed between the two groups (*p* = 0.7173) (Table 2).

**Table 2:** Comparison of treatment effectiveness between groups

	50% group (*n* = 17)	75% group (*n* = 15)
Effective	8 (47.1%)	5 (33.3%)
Slightly effective	4 (23.5%)	4 (26.7%)
Aggravated	5 (29.4%)	6 (40.0%)
*p*	0.719	

*Note*: The treatment was deemed “effective” if posttreatment AHI improved to <10 and AHI improved to 50% or higher. Furthermore, the treatment was deemed “slightly effective” if a sufficient therapeutic effect was not achieved, and “aggravated” if posttreatment AHI was higher than the pretreatment value. No significant differences in effective treatment were observed between groups (*p* = 0.719). 50% group: 50% mandibular advancement. 75% group: 75% mandibular advancement. Abbreviation: AHI, apnea‐hypopnea index.

Furthermore, the subjects whose treatment was effective were found in one of eight males and seven of nine females, slightly effective were found in two of eight males and two of nine females, and aggravated were found in five in eight males and zero of nine females in the 50% mandibular advancement group, while the subjects whose treatment was effective were found in zero of nine males and five of six females, slightly effective were found in four of nine males and zero of six females, and aggravated were found in five in nine males and one of six females in the 75% mandibular advancement group. Significant differences in effective treatment were observed between genders in both mandibular advancement groups (*p* = 0.009, *p* = 0.003, respectively) (Table 3).

**Table 3:** Comparison of treatment effectiveness between genders in two groups

	50% group (*n* = 17)		75% group (*n* = 15)	
	Male (*n* = 8)	Female (*n* = 9)	Male (*n* = 9)	Female (*n* = 6)
Effective	1 (12.5%)	7 (77.8%)	0 (0.0%)	5 (83.3%)
Slightly effective	2 (25.0%)	2 (22.2%)	4 (44.4%)	0 (0.0%)
Aggravated	5 (62.5%)	0 (0.0%)	5 (55.6%)	1 (16.7%)
	*p* = 0.009**		*p* = 0.003**	

*Note*: The treatment was deemed “effective” if posttreatment AHI improved to <10 and AHI improved to 50% or higher. Furthermore, the treatment was deemed “slightly effective” if a sufficient therapeutic effect was not achieved, and “aggravated” if posttreatment AHI was higher than the pretreatment value. There were significant gender differences in treatment effectiveness in both mandibular advancement groups (*p* = 0.009, *p* = 0.003, respectively). **p < 0.01. 50% group: 50% mandibular advancement. 75% group: 75% mandibular advancement. Abbreviation: AHI, apnea‐hypopnea index.

Treatment success (as defined by AHI < 10) was observed in 11 of 17 patients (64.7%) in the 50% mandibular advancement group, and 8 of 15 patients (53.3%) in the 75% mandibular advancement group. Treatment success (as defined by AI < 5) was observed in 12 of 15 patients (80.0%) and 10 of 13 patients (76.9%) in the 50% and 75% mandibular advancement groups, respectively. No significant difference in treatment success was observed between groups.

According to pretreatment AHI and posttreatment AHI in the 50% mandibular advancement group (*n* = 17), power (1‐β) was 0.239, using a post hoc power analysis test.

There were no patients who complained of pain in the temporomandibular joint, masticatory muscles, and lost the posterior teeth contact in both groups during 3 or 4 months.

## 4 DISCUSSION

An OA can be adjusted by the patient or the dentist, depending on the degree of subjective symptoms, such as snoring and daytime sleepiness. According to a report by Almeida et al. (2009) 60% advancement was effective for 65% of the subjects, and the effective treatment rate increased to 95.4% when the mandible was advanced further. In order to examine the therapeutic effect of OAs with a 50% or 75% advancement position as the initial therapeutic mandibular position in the present study, adjustment of mandibular advancement was not performed, and the PSG test was conducted 3–4 months after OA provision. It was desirable to compare the effectiveness and side effects of OA not only at two advancement positions but also at various advancement positions; however, an increased number of subjects would be required.

Several studies have reported that side effects of OAs, such as occlusal changes, toothache (Aarab et al., 2010; de Almeida et al., 2005; Marklund et al., 2001; Pantin et al., 1999), and temporomandibular disorders (Cunali et al., 2009; Johnston et al., 2002) intensify with an increasing degree of mandibular advancement. Since we aim to reduce the burden of treatment on OSA patients, the side effects of OAs should be kept to a minimum. On the other hand, current evidence suggests that a larger amount of mandibular advancement results in a greater improvement of OSA symptoms (Clark et al., 1993; Ferguson et al., 2006).

Rashmikant et al. (2013) found that OAs with 50% advancement or 75% advancement significantly improved ESS and sleep‐related visual analog scale (VAS) compared to maximum intercuspation, and significantly increased the pharyngeal area in the lateral cephalogram in patients with OSA. No significant difference was reported between 50% and 75% advancements. Walker‐Walker‐Engström et al. (2003) reported that there were no significant differences in AI and AHI between these two therapeutic positions in patients with severe OSA. Tegelberg et al. (2003) reported that 50% and 75% of advancements achieved a comparable rate of effective treatment in patients with mild to moderate OSA. We found that AHI improved significantly in both groups, while AI was only significantly improved in the 50% mandibular advancement group. There was no significant improvement in ESS in either of the groups.

Treatment success (as defined by AHI < 10) was documented in 64.7% and 53.3% of the patients in the 50% and 75% mandibular advancement groups, respectively. A higher rate of treatment success was observed when it was defined by AI < 5: 80.0% and 76.9% in the 50% and 75% mandibular advancement groups, respectively. This suggests that OA treatment was more effective for apnea than hypopnea. While treatment success rates were higher with 50% mandibular advancement compared to 75% mandibular advancement, this difference was not statistically significant in patients with mild to moderate OSA. Sakamoto et al. (2019) reported that 75% advancement was effective in severe cases, whereas 50% advancement was effective in moderate cases. These results suggest that the degree of mandibular advancement required for treatment success may differ according to OSA severity. Bartolucci et al. (2016) stated that providing a mandibular advancement greater than 50% did not significantly improve treatment success rates. Similarly, Tegelberg et al. (2003) have also recommended that OA treatment for patients with mild to moderate OSA should not utilize a mandibular advancement greater than 50%. Thus, for patients with mild to moderate OSA, 50% mandibular advancement should be the initial therapeutic mandibular position.

Effective treatment across both mandibular advancement groups was more often documented in females compared to males. Vecchierini et al. (2019) reported that OA therapy was effective in females with OSA of any severity, with response rates being significantly higher compared to males, especially in severe OSA. Similar to previous studies, our results also suggest that sex differences are predictors of effective treatment. Males have a longer, softer oropharynx and larger, fatter, more posterior tongue, increasing the susceptibility of upper airway collapse (Lin et al., 2008). Upper airway collapsibility, determined by the pharyngeal critical closing pressure, was found to be more in males than in equally overweight/obese and severity of OSA females (Jordan et al., 2005). At present, though it is not declared clearly, the upper airway of females might be easily enlarged by wearing OA.

According to the American Academy of Dental Sleep Medicine, “OAs should be custom‐made and allow the mandible to be advanced in increments of 1 mm or less with a protrusive adjustment range of at least 5 mm” (Scherr et al., 2014). It was reported that custom‐made OA is more efficacious in reducing OSA severity than a prefabricated thermoplastic or boil and bite OA (Vanderveken et al., 2008). As the TheraSnore™ used in this study can only be adjusted in increments of 1.5 mm, it is hardly a titratable device. If customized and titratable OA is applied to patients with OSA, treatment success might be higher than our results. Under the Japanese insurance system, the cost of this OA is approximately $227–270, whereas a fully customized and titratable OA costs approximately $2000–3000 (Levendowski et al., 2012). Thus, the high cost of fully customized and titratable OAs limits their suitability as the first choice for initial OA therapy.

This study has some limitations. The treatment effect was based on the PSG test and ESS. As the ESS was only performed on 15 subjects and the AI was taken on only 28 subjects, the results may not have reflected the trend for all patients. The value of power was 0.349, which was too low, so type Ⅱ error (false‐negative) might have occurred from these results. To achieve higher power, the sample size should be increased in our future study.

OSA is mostly found in middle‐aged men (Bixler et al., 1998) and postmenopausal women (Jehan et al., 2016). The age distribution of the patients who enrolled in this study was older. Our results cannot be generalized to other age groups.

In this study, only short‐term side effects, such as temporomandibular joint symptoms, toothache, and posterior teeth contact, were evaluated during 3–4 months. In long‐term OA use, occlusal changes, especially reduction of overjet have been reported in several studies (Malklund et al., 2019; Pliska et al., 2014). The amount of occlusion change was not related to the type of device, but rather to the duration of therapy. Long‐term several occlusal changes associated with OA use also warrant attention and need to be evaluated by future studies. Also, self‐reported questionnaire should be used to assess compliance with OA use in each group.

Moreover, while OSA patients were randomly divided into two groups, this study did not utilize a crossover design. Randomized crossover trials are required to reduce the impact of intersubject variability on the treatment outcomes.

## 5 CONCLUSIONS

In this study, patients with mild to moderate OSA were randomly allocated to receive OAs set at a mandibular advancement of either 50% or 75%. The subjects were instructed to wear the OAs, and AHI was compared pre‐ and posttreatment in both groups. AHI improved significantly in both groups, while AI only improved significantly in the 50% mandibular advancement group. No significant improvement in ESS was observed in either group. Furthermore, no significant differences in effective treatment and treatment success were observed between groups. Effective treatment was significantly more likely to be documented in females compared to males. The results of this study indicate that OA treatment for patients with mild to moderate OSA should utilize a 50% mandibular advancement as the initial therapeutic mandibular position.

## AUTHOR CONTRIBUTIONS

*Conception and design of study*: Eri Makihara and Shin‐ichi Masumi. *Acquisition of data*: Eri Makihara and Takafumi Watanabe. *Analysis and/or interpretation of data*: Hiromichi Ogusu. *Drafting the manuscript*: Eri Makihara and Shin‐ichi Masumi. *Revising the manuscript critically for important intellectual content*: Eri Makihara. *Approval of the version of the manuscript to be published*: Eri Makihara, Takafumi Watanabe, Hiromichi Ogusu, and Shin‐ichi Masumi.

## CONFLICT OF INTEREST

The authors declare no conflict of interest.

## Data Availability

The data that support the findings of this study are available from the corresponding author, Eri Makihara, upon reasonable request.
